# A Middle Palaeolithic wooden digging stick from Aranbaltza III, Spain

**DOI:** 10.1371/journal.pone.0195044

**Published:** 2018-03-28

**Authors:** Joseba Rios-Garaizar, Oriol López-Bultó, Eneko Iriarte, Carlos Pérez-Garrido, Raquel Piqué, Arantza Aranburu, María José Iriarte-Chiapusso, Illuminada Ortega-Cordellat, Laurence Bourguignon, Diego Garate, Iñaki Libano

**Affiliations:** 1 Centro Nacional de Investigación sobre la Evolución Humana (CENIEH), Burgos, Spain; 2 Department of Prehistory, Universitat Autonoma de Barcelona, Barcelona, Spain; 3 Laboratorio de Evolución Humana, Universidad de Burgos, Burgos, Spain; 4 Departamento de Cristalografía y Mineralogía, Facultad de Geología, Universidad Complutense de Madrid, Madrid, Spain; 5 Departamento de Mineralogía y Petrología, Facultad de Ciencia y Tecnología, Universidad del País Vasco/EHU, Leioa, Spain; 6 Departamento de Geografía, Prehistoria y Arqueología, Facultad de Letras, Euskal Herriko Unibertsitatea UPV/EHU, Vitoria-Gasteiz, Spain; 7 IKERBASQUE, Basque Foundation for Science, Bilbao, Spain; 8 INRAP, UMR 7041 Arscan/AnTet, Campagne, France; 9 Ramón y Cajal Senior Grant, Instituto Internacional de Investigaciones Prehistóricas de Cantabria, Universidad de Cantabria, Gobierno de Cantabria, Santander, Spain; 10 Edestiaurre Arkeologia Elkartea, Barrika, Spain; Universita degli Studi di Ferrara, ITALY

## Abstract

Aranbaltza is an archaeological complex formed by at least three open-air sites. Between 2014 and 2015 a test excavation carried out in Aranbaltza III revealed the presence of a sand and clay sedimentary sequence formed in floodplain environments, within which six sedimentary units have been identified. This sequence was formed between 137–50 ka, and includes several archaeological horizons, attesting to the long-term presence of Neanderthal communities in this area. One of these horizons, corresponding with Unit 4, yielded two wooden tools. One of these tools is a beveled pointed tool that was shaped through a complex operational sequence involving branch shaping, bark peeling, twig removal, shaping, polishing, thermal exposition and chopping. A use-wear analysis of the tool shows it to have traces related with digging soil so it has been interpreted as representing a digging stick. This is the first time such a tool has been identified in a European Late Middle Palaeolithic context; it also represents one of the first well-preserved Middle Palaeolithic wooden tool found in southern Europe. This artefact represents one of the few examples available of wooden tool preservation for the European Palaeolithic, allowing us to further explore the role wooden technologies played in Neanderthal communities.

## Introduction

The production and use of wooden tools in the European Late Lower-Early Middle Palaeolithic has been indirectly attested through use-wear analyses [1–4], but direct evidence is much more scarce, most likely due to preservational biases, and only a few sites above latitude 48 have yielded preserved wooden tools (Schöningen, Lehringen and Clacton) [5–8]. The site of Bad-Cannstatt, in Germany, has also yielded maple (*Acer campester*) fragments interpreted as tools, but theses remains were heavily altered and thus are difficult to interpret [9]. Interestingly, most of these wooden tools have been interpreted as throwing and thrusting spears. This is the case for the Lehringen spears, for the Clacton spear fragment and for most of the tools recovered in Schöningen. Most of these tools were made on *Taxus baccata* (yew, Clacton and Lehringen) and *Picea sp*. (spruce, Schöningen), with some examples on *Pinus sylvestris* (pine, Schöningen). The technology required to produce these spears was quite complex: to begin with, a long and thin shaft was selected, the bark and the knots were removed and the point, usually placed away from the central axis of the trunk, was obtained through scraping and polishing, maybe aided by fire [10]. Some authors have argued that this kind of tool production represents a significant cognitive leap for hominids because the complexity involved in the process implies abstraction and in-depth planning capacities [11]. Others, on the other hand, have argued that the wooden tool-making process might have been far more simple than is currently thought [12]. Besides, other kinds of wooden tools have been also identified, but are less abundant, among them the pointed stick from Schöningen [6]. In southern Europe the only direct evidence of wooden tools predating modern human arrival are the wooden artefacts from Abric Romaní and the recently discovered sticks from Poggetti Vecchi. At Poggetti Vecchi more than 30 fragments interpreted as sticks have been recovered from a MIS7-6 open air context [13, 14]. At Abric Romaní putative tool functions have been inferred from the morphologies of the wooden artefacts, including objects interpreted as vessels or shovels [15–16. One single wooden pseudomorph from Abric Romaní level J has been interpreted as a possible digging stick or fragment of a stake [17].

Here we present a wooden pointed tool found at Aranbaltza III (Basque Country, northern Spain) dated to the early Late Pleistocene, which represents the oldest wooden tool from southern Europe, in this case associated with Neanderthals.

## Archaeological setting

**Fig 1:**
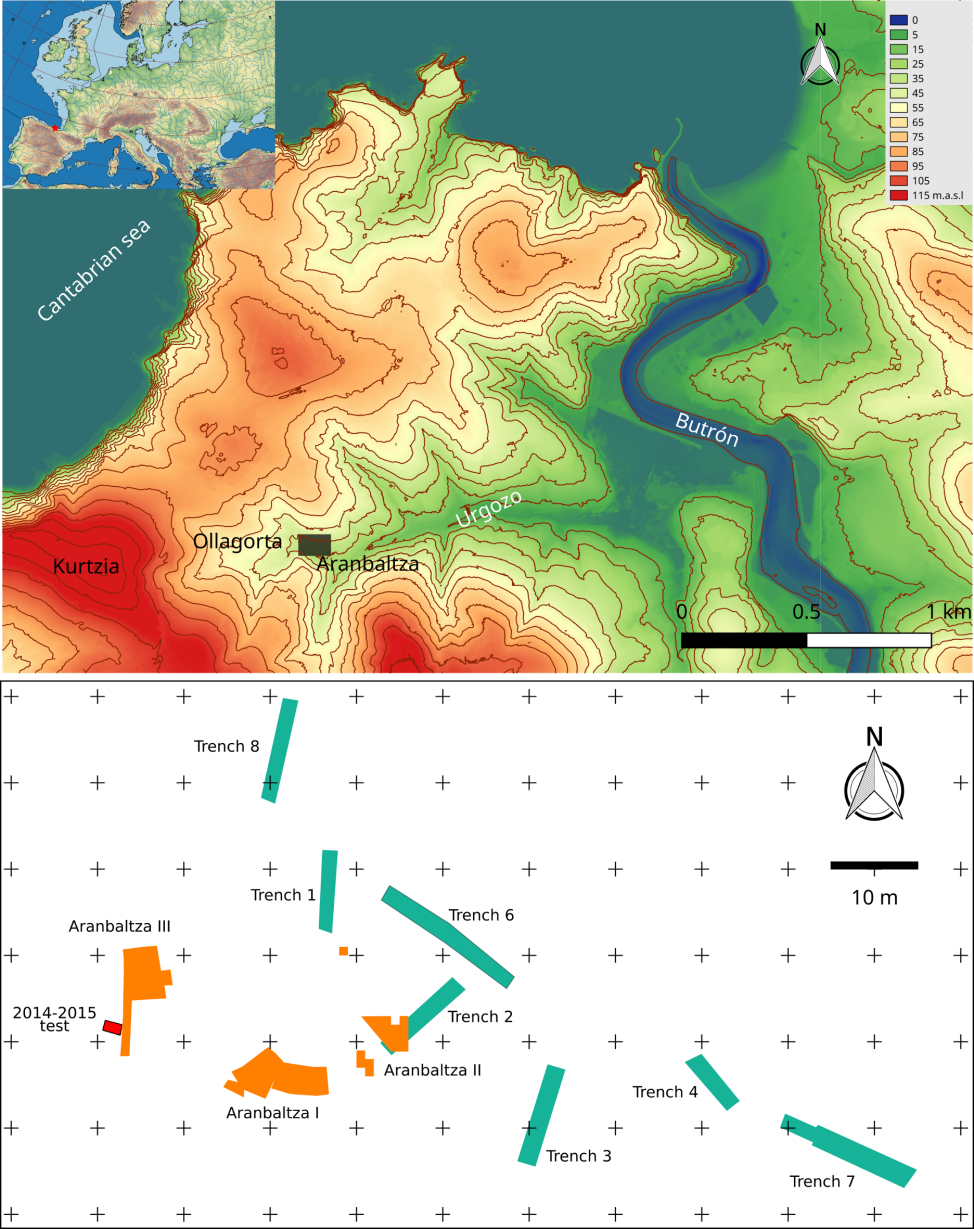
(top) location of the Aranbaltza archaeological complex; (bottom) position and extension of the different excavation areas. Raster data obtained from Eusko Jaurlaritza / Gobierno Vasco. GeoEuskadi and from the European Environment Agency. Rivers and bathymetry vectors obtained from Natural Earth. Map elaborated with QGIS 2.8 Wien and Inkscape 0.91.

The site of Aranbaltza is located in the coast of Basque region, close to Bilbao. The site is situated in the bottom of a small valley that runs towards the Butron river-mouth. Although the current coastline is very close to the site (800 m NW) it is separated from the site by a raised cliff (90 m.a.s.l). The site was discovered in 2004 [18], close to the site of Ollagorta, where in 1959 J.M. Barandiaran excavated several test-pits in the front area of a sand quarry [19]. Since 2013 archaeological excavations at the Aranbaltza complex have identified three archaeological sites (Aranbaltza I, II and III) (Fig 1) with comparable archaeo-sedimentary sequences spanning from the Late Middle Pleistocene all the way to the Holocene.

**Fig 2:**
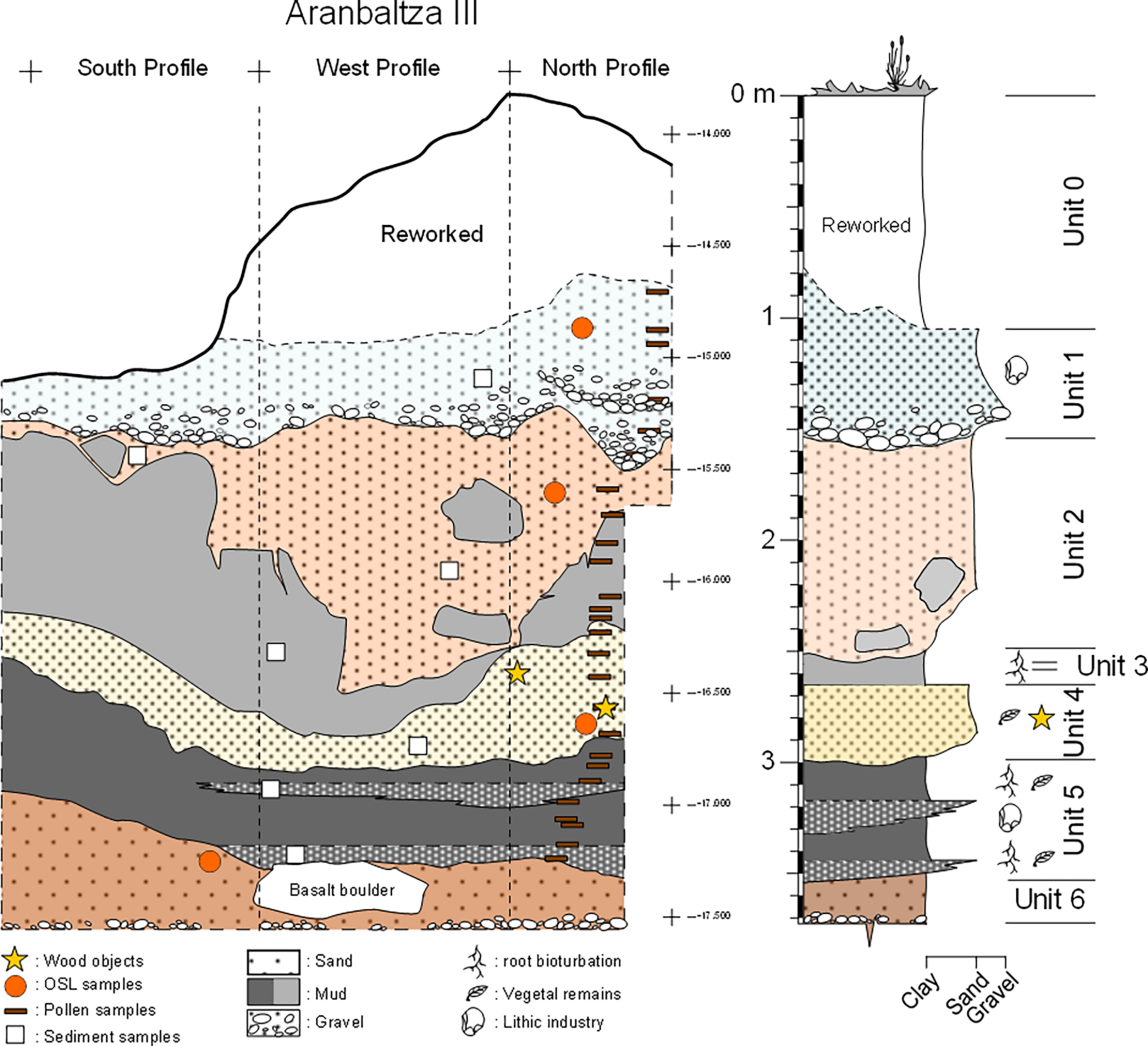
Lithostratigraphic panel and synthetic stratigraphic column of the Aranbaltza III site. The locations of wooden remains, OSL, pollen and sedimentological samples are noted.

At Aranbaltza III a 2m^2^ test pit was excavated between 2014 and 2015 (UTM 30N x: 502713.6, y: 4805178.6, z: 37). As part of this excavation, a total of six lithostratigraphic units and four sedimentary facies were defined (Fig 2) (S1 File). From top to bottom:

- **Unit 0** represents modern reworked sediments.
- **Unit 1** is a channel sandy infill with a basal lag where abundant Mousterian lithic remains were found (S2 File).
- **Unit 2**, which is archaeologically sterile, has been interpreted as representing an incised channel infill consisting of multiple sandy high density flowing events, extensively altered by edaphic processes (podzolization).
- **Unit 3** is made up of bioturbated floodplain clays and, as the previous unit, is archaeologically sterile.
- **Unit 4** is a thick sand deposit interpreted as representing a tractive sandy sediment sheet formed in a crevasse splay/channel; In this unit two wooden tools and a single flint tool (see S2 File) were recovered. One of the wooden tools is the point described here (S5 File), the other one is a fragment of a pulled out branch that preserves part of the ripped joint with the parent trunk, having the distal end intentionally pointed (S6 File). Both tools were found in almost vertical position inside the sandy sediment, and both of them bear abrasion traces caused by sand in movement, as does the flint tool recovered with them. This suggests that the pieces were not *in situ* but reworked from stratigraphically older lateral deposits (probably Unit 5).
- **Unit 5** consists of interbedded decimetre-scale layers of grey-to-blackish sandy organic muds (subunits 5a and 5c) and clayey sands (subunits 5b and 5d) deposited in a vegetated backswamp area where sandy sediments were deposited during flood events (crevasse channels or lobules); this unit is rich in lithic artefacts (S2 File) and unworked wood remains.
- **Unit 6** is an incised channel infill corresponding to multiple sandy high-density flowing events, and it is archaeologically sterile.

Lithostratigraphical units 1, 2, 4 and 6 correspond to different types of fluvial channel infillings, the former and latter are probably laterally migrating shallow channels, while Unit 2 corresponds to an incised channel infill consisting of multiple sediment gravity flows. The lower lithostratigraphic units, (3, 4 and 5), are interpreted as representing overbank fine architectural elements, deposited in floodplain environments. The observed lithofacies were deposited in crevasse splay and backswamp environments. Units 2, 3, 4 and 6 have been dated by OSL (S3 File). Despite the problems of differential bleaching of quartz grains, the sequence can be dated confidently between ca. 137–50 ka. The Minimum Age Model for Unit 4 yielded an age of 70.0±8.4 ka.

The pollen analysis carried out for Unit 4 suggests a formation under relatively temperate and humid conditions. The dynamics of Gramineae, heathers and Compositae (main components of the herbaceous-shrub layer), alongside the diversity of hydrophyte and vascular aquatic plants (Ranunculaceae, Cyperaceae, Liliaceae, *Typha* and *Potagometon*) and bryophytes like *Sphagnum* suggest the presence of a waterlogged environment. Tree cover (circa 40%) is dominated by conifers (>70%) and mixed deciduous forest. Among the latter, *Taxus* pollen has been identified.

## Methods

The wooden pointed tool recovered at Aranbaltza III, is analyzed here. A morphological description, an anatomic and taxonomic classification, a technological analysis and a use-wear analysis will be presented.

The piece became deformed as a result of the preservation procedures, including shrinking and bending. The original morphology of the piece was reconstructed using virtual restoration procedures comprising photos of the piece obtained at the moment of its discovery and the surface scans made taken with an Artec Spider scan (S4 File, S5 File). The morphological and metric description was made using this restored model and the actual piece, which, thanks to the preservation techniques employed, shows very well preserved surfaces.

The external morphology and the internal structure of the piece were analyzed in order to classify it anatomically. The internal structure analysis was carried out by means of a MicroCT scan of the piece. The sample was scanned using a 240 kV X-ray tube working at 50 kV and 100 μA, producing 1200 radiographs at a 27 μm resolution.

For the taxonomic classification, a small splint of wood was extracted from the surface that was damaged during excavation, and the identification was made through its microscopic analysis and comparison with a reference atlas [20].

For the technological analysis, the morphometric and anatomic features were considered (for example the position of the central pitch), and the technological wear observed on the surface was characterized and compared against already-available descriptions [6, 8] and experimentally-reproduced wooden tools [21]. The undertaking of a high power use-wear analysis was not possible due to preservation issues, but a low-power analysis was carried out in order to obtain direct insights on tool-function [21].

## The wooden pointed tool

**Fig 3:**
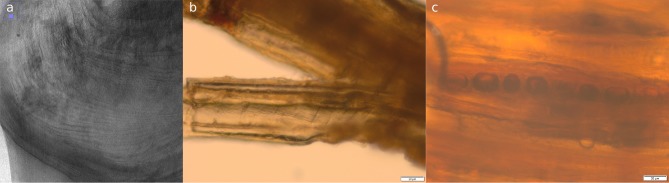
Tree species identification. a) CT scan image of the transversal cross-section; the distinct growth rings of a coniferous wood can be seen. b) Longitudinal-tangential cross-section, rays between 6 and 11 cells high can be distinguished. c) Longitudinal-radial cross-section, spiral thickenings on the tracheid walls can be observed.

The point was recovered during the section cleaning of Unit 4 and, as a result, suffered some damage to one of its sides. The pointed tool was initially photographed and then stored in a watertight container alongside the original sediment in which it was found. The microscopic analysis of a small fragment recovered from the damaged area has allowed us to identify the tree species from which it was made: yew (*Taxus baccata*) (Fig 3). The presence of this species in the surroundings of the site was also identified in the pollen analysis. Although this species has rarely been identified in Pleistocene archaeological contexts [22], it was used to make the spears found at Clacton-on-Sea and Lehringen [5, 6]. In the Iberian Peninsula only a few yew charcoal remains have been identified for this period, at the Middle Palaeolithic site of Can Costella [23] (northeast Iberian Peninsula). Yew is highly appreciated in woodworking because its wood is hard, flexible and rot-resistant; it has been used traditionally to make spears and bows [24].

**Fig 4:**
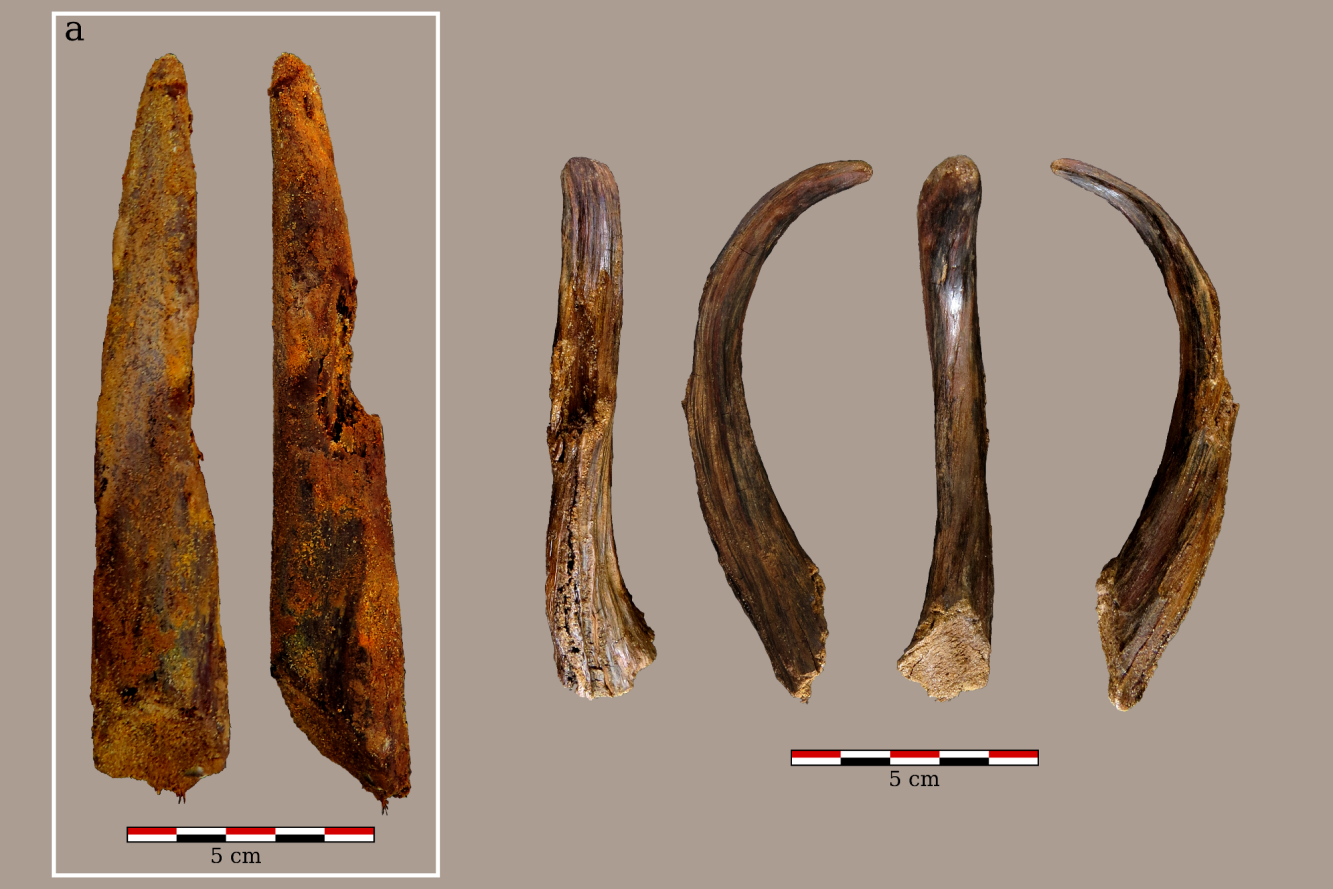
The wooden pointed tool. a) Photograph showing the pointed tool immediately following its recovery. b) Current appearance of the point fragment following preservation efforts.

**Fig 5:**
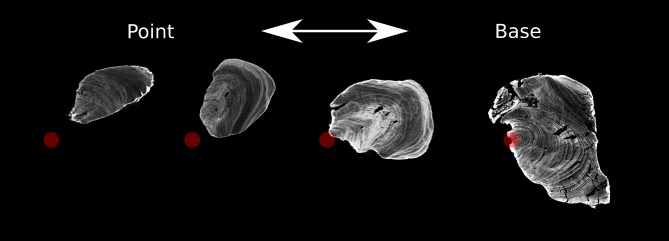
Selected MicroCT slices. The red dots indicate the actual or estimated position of the centre of the branch.

The original morphology of the piece was a straight stick 151.7 mm in length and 28.6 mm in width, with an irregular or slightly oval transversal section, with a pointed distal end (rounded U-shape) and a beveled proximal part (Fig 4). One third of the surface corresponds to the sub-cortical part of a big branch, with small twig knots on it. The growing direction of the twigs is towards the pointed end indicating that the beveled end was the closest to the roots. The internal structure of the point, more precisely the growing rings, reveals that towards the base the centre of the branch is located close to the lateral surface, while the point is placed far away from the central axis of the branch (Fig 5). Half of the diameter of branch is preserved at the base, while only a fraction of it is preserved in the point area. The piece does not show bark or inner bark.

**Fig 6:**
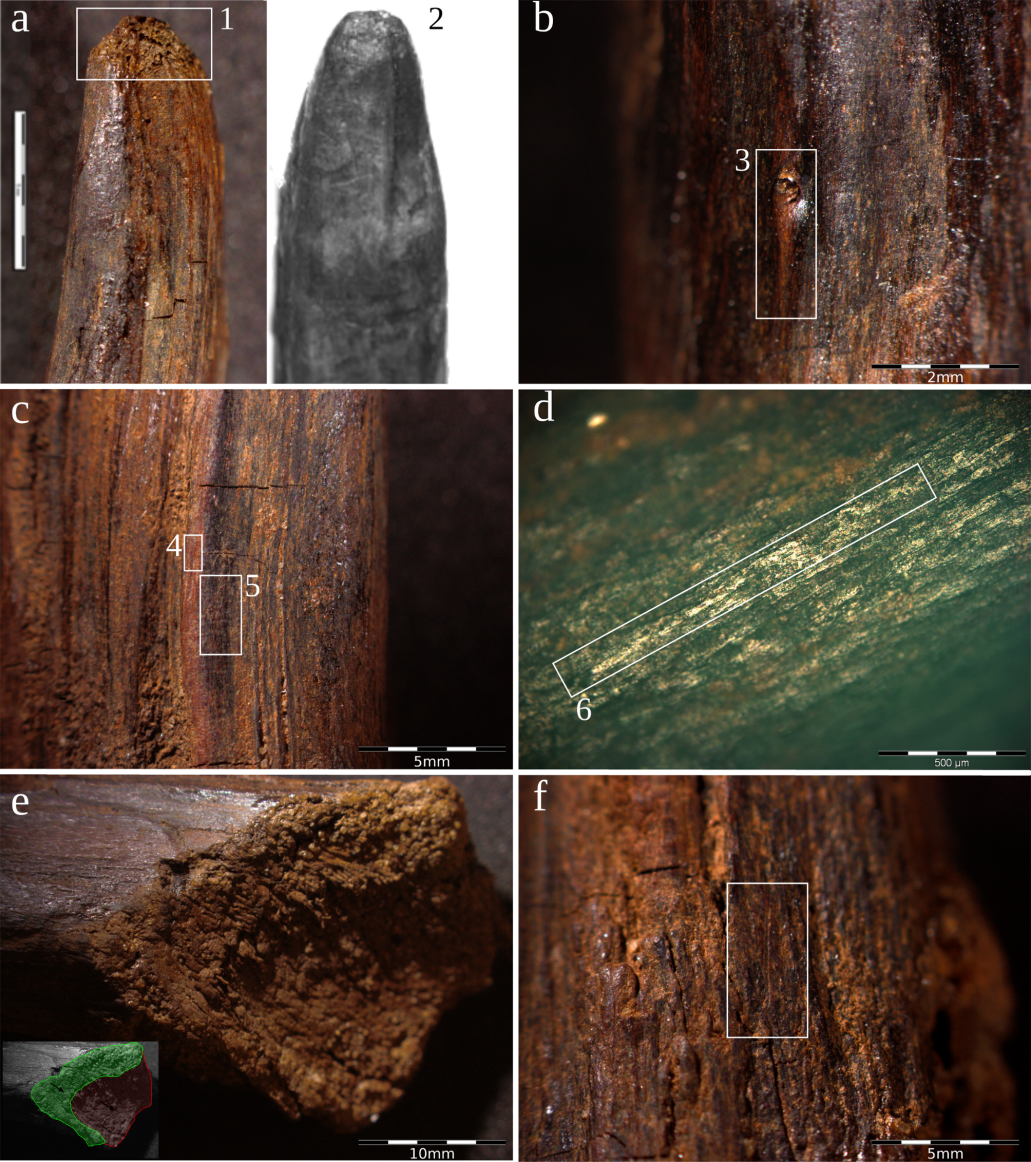
Technological and use wear features. a) Detail of the smashed fibres in the point 1: Aranbaltza pointed stick, 2: experimental pointed stick with smashed fibres on its pointed end [21]; b) detail of the pulled-off twig knot; c) detail of the thermal alteration 4: red colouration, 5: black colouration; d) microscopic detail of the polished surface; e) detail of the beveled end with the two plans corresponding to the two chopping strokes; f) small cut mark on the wood surface.

The surface shows little technological evidence of chopping and shaping (Fig 6). Only two small cut-marks, corresponding with the latest phases of shaping, have been noted on the surface. The twig knots do not show any wear linked to trimming using a cutting edge, thus we could interpret these as having simply been pulled off (Fig 6B). The surface is polished (Fig 6D), which could probably explain the absence of other manufacture traces; nevertheless we were not able to rule out the possibility that this polish was the result of an alteration caused by the contact with the sandy sediments of Unit 4. Also, the surface shows important colouration changes, with reddish and blackish tones (Fig 6C), suggesting that the point underwent a thermal alteration, maybe as a result of its hardening and/or shaping through the use of fire [10, 14]. The bevel on the proximal end reveals a rough surface, with exposed fibres and two different planes, suggesting that it was chopped by means of two strokes (Fig 6E and 6F). The surface of the bevel does not show traces of polishing. These differences in the surface could suggest that the bevel was shaped after the point was finished and used. This would mean that this is a recycled tool fragment, but we cannot rule out that this simply represents a different technological treatment of different parts of the same tool.

Finally, the point shows that the smashed fibres on its end were caused by some kind of repetitive mechanical stress only affecting that particular area (Fig 6A1). This kind of wear has been identified on Neolithic wooden pointed digging sticks at the site of La Draga [18], and the experimental replicas of these digging sticks demonstrate that this kind of use-wear is produced when removing medium-to-hard soil (Fig 6A2). Similar use-wear has also been recorded on the wooden tools identified as digging sticks at the site of Border Cave (South Africa) [25]. The Aranbaltza point tip also has a rounded (U-shaped) morphology, while the spear points recovered from Middle Pleistocene sites in Northern Europe have sharper (‘pointed’) tips. Thus, the morphology and the use-wear of the Aranbaltza point suggests that it was used as a digging stick.

Although the preservation of the piece does not allow for a precise description of the operational sequence to be put forward, we are able to identify a combination of operations, including branch shaping, bark peeling, twig removal, shaping, (probable) polishing, thermal exposition and chopping. The available evidence suggests that, as was the case with the Schöningen and Lehringen spears [6, 8], a whole branch or a thin trunk was used and the point end was placed away from the central axis. Evidence of use has also been identified suggesting that this artefact was used in a mechanical activity, like digging. Due to the short length of the point the idea that the preserved point is a recycled fragment of a bigger tool cannot be ruled out.

## Discussion

The finding of wooden artefacts in Pleistocene archaeological sites in Europe is exceptionally rare. Up to now few well-preserved artefacts have been recovered from Middle Pleistocene deposits in Northern Europe, and few more from Upper Pleistocene sites [5–8, 14– 16]. The preservation in Aranbaltza III was favoured by the formation of a waterlogged reducing depositional environment due to the rapid sedimentation of organic-rich sands and clays in a floodplain swampy area. The wooden point fragment was found in a sandy and slightly tractive deposit (Unit 4), and was probably reworked from nearby exposed underlying sediments from Unit 5, where favorable conditions for wood preservation and the presence of wooden remains have been confirmed. The age of these deposits ranges between 58–137 ka, this apparently age incertitude can be explained by the insufficient bleaching of quartz sand grains due to the nature of their sedimentary processes, which implied quick erosion and deposition at a short distance from the original sediment. The age obtained from the sandy sediments contemporaneous with or younger than Unit 5 in another sector of Aranbaltza III was ca. 90 ka, which could be interpreted as the most probable age for the pointed tool. The lithic assemblages recovered from Units 4 and 5, albeit scarce, point clearly to the Middle Palaeolithic with Discoid technology. Middle Palaeolithic occupations in the region are known since MIS7-6. Several sites have occupations dated to the MIS5-4 interval, with Arlanpe, Lezetxiki or Askondo as the most relevant [26–28]. The Early and Late Middle Palaeolithic in the region are characterized by great behavioural variability; the long-distance transport of lithic raw materials [29], the trend towards microlithization [30], the use of complex hunting technologies [31], the fire control and use [32], use of bone tools [33, 34], a certain degree of prey specialization [35] or the exploitation of marine resources [36] being especially remarkable. We should now add elaborate wooden technology to this behavioural complexity, drawing a picture of well-adapted and flexible Neanderthal populations in the region.

The oldest wooden tools, recovered at Schöningen and Clacton, are associated with Lower Palaeolithic industries, and reveal an early use of wooden artefacts for hunting and other activities [5–7]. The wooden sticks from Poggetti Vecchi have been dated to MIS7-6, and thus can be also associated to Neanderthals. The function of these objects, inferred from their morphologies, has been interpreted as digging sticks [14]. The spear from Lehringen was recovered in a sedimentary deposit dated to ca. 125.000 BP, and thus, is associated with Neanderthals [8]. This tool has been interpreted as a thrusting spear, similar to the spear VI from Schöningen, while other spears from Schöningen have been interpreted as throwing spears [6]. At Abric Romaní several wooden pseudomorphs were reported in levels H, I, Ksup and M, dated to the Late Middle Palaeolithic [15–16, 37].

The few available direct and indirect lines of evidence suggest that wood played a significant role in Neanderthal technological adaptations. Wood provides enough plasticity to shape a varied array of tools that are impossible to obtain through the use of stones, and very difficult to obtain with bones, which have constrained sizes and are more difficult to work. The use of bone technology by Neanderthals has been widely demonstrated, but the extent of activities identified is, up to now, very limited and linked to domestic activities (polishers, chisels, retouchers) [33–34, 38–43]. Wood was surely used for manufacturing hunting weapons and as fuel [37, 44]. Other functions, as containers, hammers, or shelter construction materials, should not be ruled out even if the evidence is very scarce or completely absent [15, 45]. Different stone-tool types that have woodworking-related use-wear or tools that must have necessarily been used with a wooden haft (e.g. stone spear-points) are indirect evidence of wood use in the past [30, 46–52].

This paper has presented a new Middle Paleolithic wooden tool. The shape of this tool and the evidence of use suggest its function as digging instrument. Digging stick is a common tool in hunter gatherer societies, being root digging one of the main functions [53–56], although other uses as loosening bark or clam-digging have been also recorded.

The variability in shape and dimensions in archaeological and ethnographical digging sticks is enormous. According to Oswalt [55] digging-sticks are multi purpose tools and probably this explains the high morphological variability of ethnographic digging sticks. For example, the measures of ethnographic materials from Australian collections recorded by Nugent ranges between 420x30 mm to 1684x33 mm [57]. Similar variability has been recorded in ethnographic databases [58–59].

The length of Aranbaltza point is short compared with most of ethnographic digging sticks recorded, but it’s not rare. Different examples of short (less than 30 cm length) digging sticks can be found in ethnography [58, 60–61] as well as in archaeology [25, 62]. Besides, the Aranbaltza point displays cutting marks at the opposite end of the tip, showing the possibility of having been shortened. For this reason it can’t be discarded the possibility that the original length of this point was longer. Other wooden tools from Middle Paleolithic sites have been also interpreted as wooden sticks. A pointed wooden pseudomorph from Abric Romaní’s level J (ca. 50 kyr) has been interpreted, based on its morphology, as a massive digging stick, or more probably as the end of a post or a stake, but no direct use-wear evidence is available [17]. Also, the tool fragments from Poggetti Vecchi have been interpreted from its morphology as digging sticks [14].

Digging soil can be done for different reasons, for finding edible USOs (Underground Storage Organs- tubers and roots) or animals; for extracting lithic raw materials or for making negative structures (i.e. pot holes or sepultures). In the archaeological record there is almost no empirical evidence to support this kind of activities, which have been inferred through indirect evidence. For example, the consumption of USOs by Neanderthals in Europe has been suggested through direct analysis of dental calculus or fecal remains [63–65]. In the surroundings of Aranbaltza different edible USOs would have been available given their known distributions in different climatic scenarios [66]. Furthermore, the pollen analysis from Unit 4 revealed the presence of cattails (Typha), indicating that plants with edible USOs could be found close to the site. On the other hand, there is little evidence of underground animal gathering through digging [67], but the presence of hare and rabbit has been documented in contemporary sites like Axlor, Lezetxiki, Covalejos or Atxagakoa [68–70]. Also, shellfish gathering has been proposed as a possible function for digging sticks. Although no direct evidence is available at the site, in the nearby site of El Cuco, dated back ca. 44 ka BP [36] unquestionable evidence of limpet consume has been recorded. Regarding raw material collection, the presence of flint from primary sources has been attested in different levels of Aranbaltza III, and also in the Middle Palaeolithic levels from Aranbaltza I. Flysch flint appears in different contexts close to the site (<500 m). Interestingly, the best quality flint is nowadays present in a muddy olitostrome [71] and some digging was probably needed to extract the nodules. Finally, Neanderthals are known to have dug simple structures into the soil to build shelters [45] or to bury corpses [72]. In Aranbaltza I, remnants of stone structures (pavements, fireplaces and windshields) built by Neanderthals have been found in association with abundant lithic remains probably contemporaneous with Unit 1 from Aranbaltza III. For this kind of construction to take place a certain degree of digging must have been needed.

## Conclusion

A well-preserved Middle Palaeolithic wooden tool has been recovered in southern Europe. The analysis of the technological features and the use-wear of the artefact have revealed that it was shaped from a yew trunk through a complex operational sequence to create a pointed tool that was used as a digging stick. This one of the first evidence of such a tool in a Late European Middle Palaeolithic context and its possible functions have been explored, including its use in the procurement of USOs, burrowing animals, and/or lithic raw materials; or to dig features in the soil. This is one of the rarer examples in which we are able to delve directly into Palaeolithic wooden technology thanks to the particular and exceptional preservation conditions of this piece. This artefact highlights the relevance that wooden technology must have had for Neanderthal communities, a relevance that has been perceived almost always through indirect sources of evidence.

## Supporting information

S1 File Stratigraphy and sedimentology of Aranbaltza III sequence. (PDF)

S2 File Lithic assemblages from Aranbaltza III sequence. (PDF)

S3 File OSL dating. (PDF)

S4 File **Virtual reconstruction of the pointed tool (right) obtained from 3D model of the piece at its current condition (left)**.
(TIFF)

S5 File 3D reconstruction of the wooden pointed tool (.obj file with texture). (ZIP)

S6 File 3D reconstruction of the other wooden tool from U4 (.obj file with texture). (ZIP)
