# Supplementary material for: A Middle Palaeolithic wooden digging stick from Aranbaltza III, Spain
Source: PLoS One. 2018 Mar 28;13(3):e0195044. doi: 10.1371/journal.pone.0195044 (PMC5874079; doi:10.1371/journal.pone.0195044)
Supplement: S1 File — (PDF) [file pone.0195044.s001.pdf]

S1. Stratigraphy and sedimentology of the Aranbaltza III sequence

The observation and description of the sedimentary sequence were carried out on a 1 x 1 x 3 m archaeological excavation pit. Different lithostratigraphic units were differentiated based on their differing sedimentary facies (Miall, 1996). Within the sediments, it was possible to define a number of architectural elements given their distinct facies assemblage, internal geometry and external form. These architectural elements are here considered with caution since the identification of large scale geometries generally requires better exposure and more data than the analysis of traditional lithofacies

Stratigraphy and sedimentology

Six lithostratigraphic units and four sedimentary facies were defined in the Aranbaltza III sequence:

Unit 0: This surficial unit is composed of mixed angular clay and sandy sediment fragments. The sediments identified in the clasts derive from underlying stratigraphic units. Unit 0 represents an accumulation of reworked sediments as a result of different anthropic excavation activities related to the exploitation of an ancient sand quarry.

Unit 1: is made up of *ca*. 50 cm-thick grey quartz sand layer with mainly scattered medium-sized chert gravels (< 10 cm) at its base. The most common facies in this unit is a massive medium-to-coarse sand, sometimes pebbly with a *Gh* type clast supported and crudely bedded by gravels at the base of the unit - *Ss* facies (Miall, 1996). No sedimentary structures are visible. The mineralogical composition of the sediments shows its main components are quartz grains (71%) and feldspars (20%), with the presence of a minor clay mineral fraction noted in the matrix (9%) (S1 Table). The basal contact is nearly horizontal, but also irregular and erosive, defining broad shallow (< 20 cm) scours in the underlying sediments from Unit 2. Unit 1 is interpreted as probably representing a laterally migrating shallow channel infill with a basal lag.

Unit 2: this unit comprises medium-to-coarse, massive-to-faintly-laminated quartz sand - *Sm* facies (Miall, 1996). No sedimentary structures are visible, but the sand lithosome embeds decimetre-scale mud intraclasts derived from the underlying clay unit (Unit 3). Its thickness is highly variable laterally, from 1 m to 10 cm. Orange and black stained areas are present near the basal contacts and around the muddy intraclasts linked to the podsolization processes. The mineralogical analysis of the sand shows it to to be composed of monomictic quartz (100%). The basal contact is highly erosive giving rise to a one-metre deep erosive channel that has been nicely preserved in the clayey sediments. Unit 2 is thought to be an incised channel fill consisting of multiple sandy high-density flowing events. It is worth noting the post-

sedimentary edaphic activity, orange and black staining, and the absence, probably related to the meteorization, of other mineral components (e.g. feldspars) found within this units, which suggests a high degree of lixiviation and illuviation (podsolization).

Unit 3: this unit comprises massive green-to-bluish-grey clays - *Fsm* to *Fr* facies (Miall, 1996). Horizontal lamination and scarce orange and black mottles, vegetal organic matter fragments and possibly root remains, are all visible in this unit. Centimetre-scale sandy layers are present towards the base of the unit, forming an irregular but quite horizontal unit bottom. Unit 3 represents the deposition of bioturbated backswamp clays on a floodplain area adjacent to a sand-rich fluvial channel.

Unit 4: this unit is made up of medium massive quartz sand - *Sm* facies (Miall, 1996). It is *ca.* 30 cm thick. Orange and black stained areas are present near the contacts linked to podsolization processes. The mineralogical study of the sands shows them to, be monomictic and 100% composed of quartz. The basal contact is irregular but quite horizontal. Unit 4 is interpreted as representing a tractive sandy sediment sheet. The broad and shallow geometry of the lithosome and its stratigraphic position between a number of floodplain deposits could be suggesting that these sediments originated from a crevasse splay/channel. It is worth mentioning the presence of post-sedimentary orange and black staining and the absence, probably related to meteorization, of other mineral components (e.g. feldspars) suggesting its podsolization.

Unit 5: this unit consists of interbedded decimetre-scale layers of grey-to-blackish sandy organic muds (subunits 5a and 5c) and clayey sands (subunits 5b and 5d) - *Fsm* and *SS* facies (Miall, 1996). Both clay and sandy units contain abundant centimetre-to-decimetre-scale plant remains. The sandy 5b and 5d units pinch out laterally and show channeliform geometries; the sands in subunit 5d are pebbly at the bottom where a large tabular volcanic clast (> 40 cm) is found. The sediments of Unit 5 were likely deposited in a vegetated backswamp area where fine-grained (clay and silt) deposits and sporadically distal crevasse splay sandy sediments (subunits 5b and 5d) also accummulated (crevasse channels or lobules) during flood events.

Unit 6: this unit comprises medium-to-coarse massive-to-faintly-laminated quartz sand - *Sm* facies (Miall, 1996). No sedimentary structures are visible. Its minimum thickness is laterally highly variable, from 20 cm to 50 cm. Orange and black stained areas linked to podsolization processes are found near the basal contacts. The mineralogy of the sands shows a monomictic quartz composition. The basal contact is not visible but it includes an abundant number of centimetre-scale quartz gravels and a thick basalt boulder. This unit is interpreted as representing an incised channel fill consisting of multiple sandy high-density flowing events. It is worth noting the evidence for post-sedimentary edaphic activity; the presence of orange staining suggests a high extent of podsolization.

Architectural elements

The architectural elements are characterized by a distinct facies assemblage, the classified based on the nature of bounding surfaces, external geometry, scale, lithology, internal geometry, and palaeocurrent patterns (Miall, 1985).

In the case of the studied sequence channel (CH) and overbank fines (FF) represent the two main architectural elements identified. Lithostratigraphical units 1, 2, 4 and 6 represent different types of channel infillings, the former and the latter are probably shallow laterally-migrating channels, and so could also be representing a lateral-accretion macroform (LA) unconformably deposited above Unit 2. Unit 2 corresponds to an incised channel infill consisting of multiple sediment gravity flows. The contrasting degree of podsolization of both units points to the presence of a sedimentary hiatus between them.

The lower lithostratigraphic units (3, 4 and 5) are interpreted as overbank fine (FF) architectural elements, deposited in floodplain environments. The observed lithofacies were deposited in crevasse splay and backswamp environments.

Although additional lateral information would be necessary, the vertical sequence ranging from thick overbank facies to shallow channel environments could suggest an aggradational stacking of progressively lower flow fluvial environments, where floodbasin sediments became less abundant and channel geometries evolved and became smaller in size.

Sedimentary environments and processes

The archaeologically-significative units 4 and 5 are interpreted as comprising backswamp sediments deposited in floodplain environments. A floodplain is the strip of land that borders a river channel, which is normally inundated during seasonal floods. Floodplains contain active and abandoned channels and bars (the channel belt), levees and crevasse channels and splays. Levees are discontinuous, wedge-shaped ridges around active and abandoned channels. Levees commonly have channels cut into their surfaces. The larger ones are termed crevasse channels (Unit 4) and split downslope into smaller distributaries surmounting fan- or lobe-shaped mounds of sediment called crevasse splays. Insofar as crevasse channels operate only during floods, they are ephemeral channels. Permanent marshes (Unit 5) may be present in wet climates.

Sediment is transported over floodplain as bed load and suspended load during overbank floods. The sources of sediment are the main and tributary channels, the valley sides and the floodplain itself. A large range of sediment sizes is commonly available, from mud to gravel. Although the grain size of sand on floodplain surfaces tends to decrease the further away one is from the active channel belt, mud usually accumulates as a more or less continuous blanket. Erosion occurs where flow is accelerated in locally narrow or topographically high floodplain sections and where vegetation cover is poor.

The basic sedimentation units in floodplains from Unit 5 are millimetre-to decimetre-thick stratasets deposited during overbank flooding events. Basal erosion surfaces are present if erosion preceded deposition. Grain sizes and internal structures depend on local flow conditions and sediment availability. The upper parts of these stratasets are commonly bioturbated with root casts. Layers of drifted vegetation are common in the overbank deposits of humid climates.

Flood-generated stratasets of crevasse splays are similar to those of levees, but channel-bar and channel-fill deposits as inferred for Unit 4 are common in crevasse splays, and these may be difficult to distinguish from the main channel deposits. The crevasse channel of Unit 4 at the site of Aranbaltza developed from the eroding floodplain marshy facies in Unit 5, resedimenting sediments and wood remains from Unit 5, including the archaeological remains described in this work.

References

Bridge, J.S. & Demicco, R.V. (2008). Earth Surface Processes, Landforms and Sediment Deposits. 815 pp., Cambridge, New York: Cambridge University Press.

Miall, A.D. (1985) Architectural element analysis: a new method of facies analysis applied to fluvial deposits. *Earth Science Reviews*, 22, 261-308.

Miall, A. (1996). The Geology of Fluvial Deposits. Sedimentary Facies, Basin Analysis, and Petroleum Geology. xvi + 582 pp. Berlin, Heidelberg, New York, London, Paris, Tokyo, Hong Kong: Springer-Verlag.

Table 1. Sedimentological, mineralogical and geochemical characteristics of the Aranbaltza III sequence.

Sedimentary unit			Unit 1	Unit 2	Unit 3	Unit 4	Unit 5a	Unit 5b	Unit 6
Depth (cm)			90-160	160-250	150-280	250-300	300-350	320-355	>310
Horizon (soil) (FAO)			Abic (Podzol)	Spodic (Pozol)	Vertic (Gleysol)	Spodic (Pozol)	Vertic (Gleysol)	Fluvisol	Spodic (Podzol)
Genetic interpretation			Fluvial channel	Fluvial channel	Backswamp	Crevasse splay	Crevasse channel	Backswamp	Fluvial channel
Colour (Munsell)			10R 5/1 10B	5R 5/8	7/1 5B	10YR 8/8	10YR 5/1	2.5Y 5/1	5R 5/8
Granulometry			Coarse sand	Sand	Clay	Sand	Silty Sand	Silty clay	Sand
Mineralogy (DRX)	Quartz	(%)	70.92	100	53.15	100	60.73	85.38	87.92
	Feldspar	(%)	17.96	-	11.47	-	-	4.85	3.41
	Clay minerals	(%)	9.12	-	35.38	-	39.28	7.16	8.88
Main elements (XRF)	SIO2	(%)	82.35	97.46	79.87	92.01	76.3	89.41	91.56
	AL2O3	(%)	6.9	0.893	13.64	3	16.73	5.08	5
	Fe2O3	(%)	3.33	0.428	1.23	0.301	1.57	1.53	0.76
	CaO	(%)	2.26	0.328	0.77	1.77	0.592	0.143	0.197
	MnO	(%)	1.47	0.0085	0.084	-	0.0041	-	0.0041
	K2O	(%)	0.942	0.156	1.43	0.249	1.95	0.586	0.624
	MgO	(%)	0.469	0.035	0.651	0.155	0.992	0.229	0.262
	TiO2	(%)	0.41	0.098	0.848	0.204	0.639	0.358	0.295
	Na2O	(%)	0.364	0.244	0.493	0.127	0.381	0.32	0.296
	SO3	(%)	0.0448	0.0166	0.0242	0.0257	0.106	0.823	0.146
	P2O5	(%)	0.0344	0.244	0.0258	1.24	0.0453	0.0182	-
	ZrO2	(%)	0.0188	0.0067	0.0368	0.0097	0.0266	0.0266	0.017
