# Supplementary material for: A Middle Palaeolithic wooden digging stick from Aranbaltza III, Spain
Source: PLoS One. 2018 Mar 28;13(3):e0195044. doi: 10.1371/journal.pone.0195044 (PMC5874079; doi:10.1371/journal.pone.0195044)
Supplement: S2 File — (PDF) [file pone.0195044.s002.pdf]

S2. Lithic assemblages from the Aranbaltza III sequence

Unit 1

The clast lags excavated in Unit 1 mainly comprise lithic artefacts, mixed together with natural flint and sandstone fragments (Table 1). Although altered by transport and atmospheric exposure, most of the assemblage is well preserved. In 2014, the whole of Unit 1 was excavated, whereas the following year three sub-units were differentiated. There are some minor differences between these sub-units, but the basic features of the three lithic assemblages remain very similar, so for this reason we have considered all the material from Unit 1 as a single assemblage. We are of course aware of the transported nature of some of this material, but there is no evidence of any significant heterogeneity. The sediment was dry-screened at the site so this could explain the relative low quantity of debris.

The assemblage comprises 901 artefacts (Figure 1). The huge majority of pieces are made on ultra-local (Flysch) flint, while other materials made on quartzite, trachyte or mudstone are anecdotal. Tested cores and fully exploited flake cores are very abundant. Among them, Levallois cores (recurrent centripetal, bidirectional) are the most representative (Figure 1: 1-2), followed by Discoid (Figure 1: 3) and Multipolar cores. Cortical flakes, complete and partial, are also very abundant, suggesting the introduction of little or no exploited blocks to the site. The presence of o*utrepassé* flakes is also noteworthy. These flakes are linked to a particular development of the Levallois technique where cortical laterals are preserved and o*utrepassé* flakes are obtained from them. Among the simple flakes, some display clear Levallois features, such as prepared platforms, straight profiles, low knapping angles, centripetal negatives and relatively low thicknesses (Figure 1: 4-8). Although a thorough techno-economic analysis needs to be undertaken to fully understand the nature of this assemblage, all of these data suggest the presence of a complete Levallois *chaîne opératoire* at the site entailing the acquisition of raw materials, full débitage and transformation of blanks into tools.

Retouched tools are quite abundant (Table 2). The most abundant type are side scrapers, most of which are not extensively re-sharpened (Figure 1: 4-6). Slightly retouched flakes and natural bases are also very abundant: Local flint often appears naturally in the shape of parallelepiped fragments (tectofragments), which are usually retouched to adapt the natural shapes of the pieces or to create new functional areas. Denticulates and notches (Figure 1: 9) are also quite abundant. The presence of becs and borers is noteworthy. Some borers are quite small, while some becs are quite large. Other tools appear less frequently; for example, backed knifes are quite atypical in the assemblage, although one of them could be classified as an Abri Audi knife. End-scrapers, burins, truncations and splintered pieces are also atypical and cannot be confused with true Upper Palaeolithic tools. There are also some flakes (Levallois flakes N=8; Levallois points N=1; Pseudolevallois points N=4), none of which are retouched. The presence of asymmetrical flakes with natural backs is also quite remarkable (N=16). Lastly, there are six pebbles, five of which are made on quartzite and one on mudstone. They all bear percussion marks and therefore can be classed as hammers. Three other small pebbles, one in sandstone and two in quartzite, also display percussion marks and can be interpreted as representing retouchers or striking platform preparers.

The features of the lithic assemblage recovered in Unit 1 of Aranbaltza III are very similar to those excavated at Aranbaltza I (Units D and E), and fit perfectly well with a Late Middle Palaeolithic open-air occupation scenario. It is difficult to compare the lithic assemblages obtained from open-air sites with the assemblages from nearby caves such as El Cuco (Gutierrez-Zugasti et al. 2017), or more distant ones like Axlor, where Flysch flint, obtained close to Aranbaltza, is quite abundant. Differences in function and raw material availability originate very different assemblages, albeit some technological features can be traced among them. At El Cuco the Late Middle Palaeolithic levels have a Levallois-based technology with a prevalent ramified microlith production. In Axlor, the lower levels have been poorly described, but also posses Levallois features (Rios-Garaizar 2012, 2017).

Table 1. Technological composition of Aranbaltza III, Unit 1.

	Sandstone	Basalt	Quartzite	Mudstone	Flint	Total
Tested cores					14	**14**
Flake cores			2		35	**37**
Levallois					*21*	
Discoid					*7*	
Multipolar					*7*	
Cortical flakes					33	**33**
Partially cortical flakes				1	109	**110**
Partially cortical blades					3	**3**
Outrepassé flakes					81	**81**
Overpassing flakes					13	**13**

Reflected flakes					4	**4**
Simple Flakes		1	2		79	**82**
Blades					10	**10**
Kombewa flakes					3	**3**
Resahrpening flakes					4	**4**
Splints					2	**2**
Irregular fragments					297	**297**
Fragments and debris <10 mm					179	**179**
Used natural bases	2		7	1	7	**17**
Flaked natural bases					12	**12**
**Total**	**2**	**1**	**11**	**2**	**885**	**901**

Table 2. Typological composition of Aranbaltza III, Unit 1.

**Bordes List Type**	**Total**
Splintered piece	**6**
Bec	**15**
Borer	**4**
Handaxe	**1**
Burin	**1**
Backed knife	**4**
Denticulate	**18**
Notch	**6**
Retouched flake and natural bases	**21**
Mousterian point	**1**
Side-scraper	22
End-scraper	**4**
Truncation	3
Raclette	**1**
Retouched tool fragment	**2**
**Total Result**	**109**

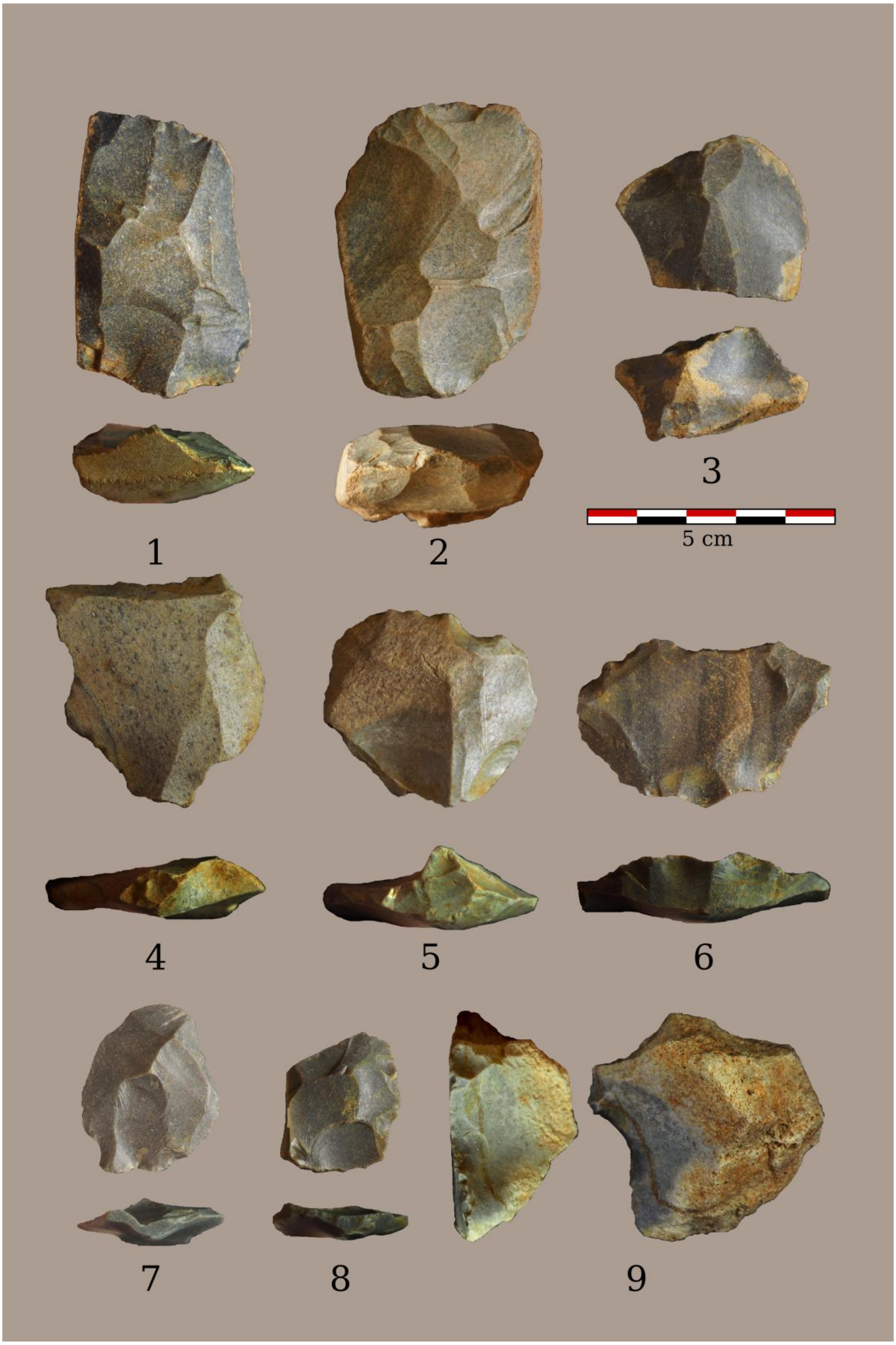

Figure 1. Lithic assemblage from Unit 1

Units 4-5

A single flake was recovered from Unit 4. It is a cortical flake with a distal non-modified concave and acute edge. It has unifacial macro-wear caused by transversal scraping or peeling (S2 Figure 2: 1).

In Unit 5 two cores and five flakes (S2 Figure 2: 2-4) were recovered, alongside several natural flint fragments, and sandstone and quartzite pebbles and fragments, probably manuports. The most interesting feature is the presence of a discoid core (S2 Figure 2: 2) and a discoid flake with use-wear traces of wood cutting (S2 Figure 2: 4), attesting to the use of discoid technology.

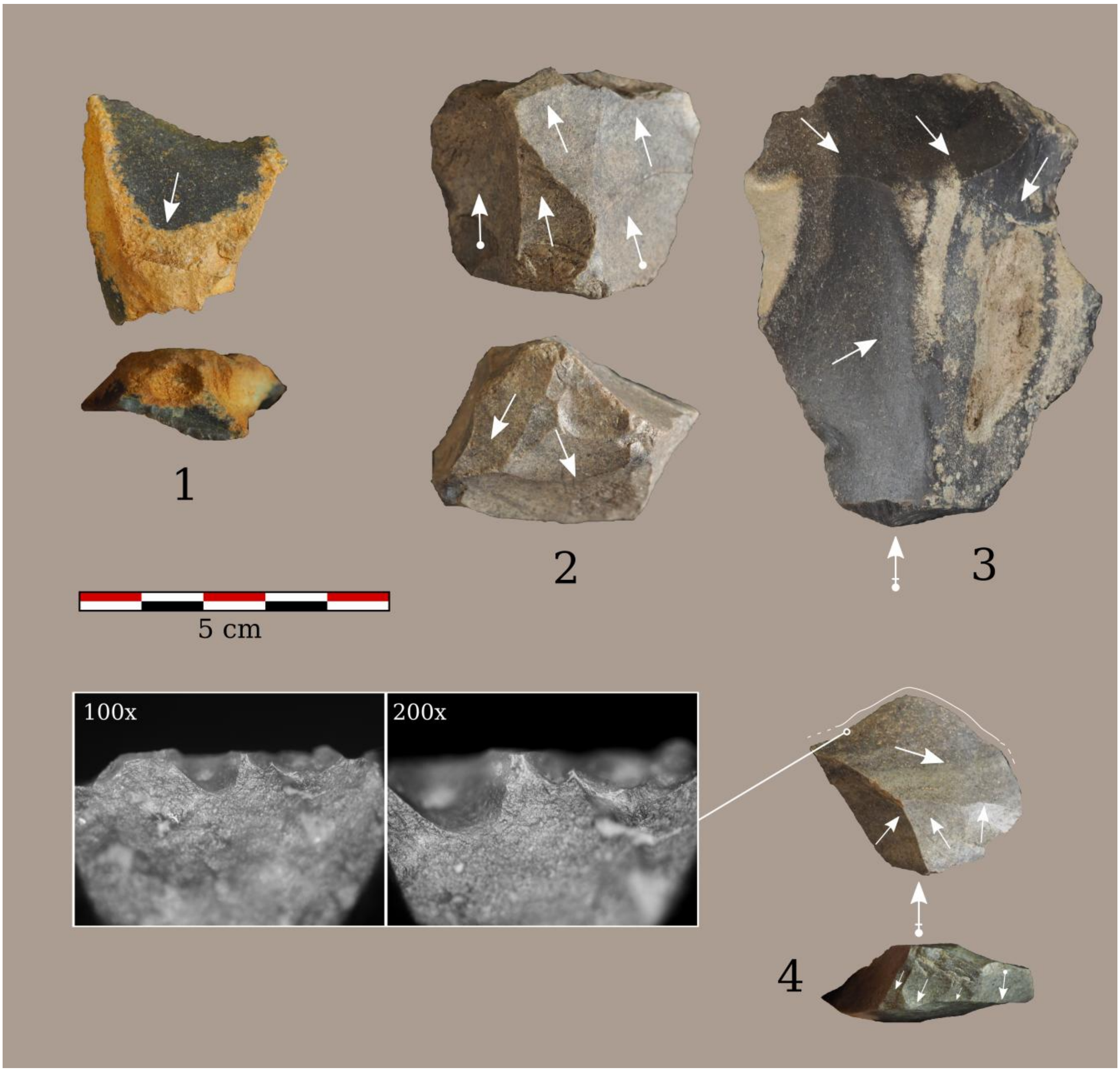

Figure 2. Lithic assemblages from Unit 4 (1), and 5 (2-4). The flake number 4 presents wood cutting wear traces.

References:

Gutiérrez-Zugasti, I., Rios-Garaizar, J., Marín-Arroyo, A.B., Rasines del Río, P., Maroto, J., Jones, J.R., et al. 2017. A chrono-cultural reassessment of the levels VI–XIV from El Cuco rock-shelter: A new sequence for the Late Middle Paleolithic in the Cantabrian region (northern Iberia). Quaternary International. doi:10.1016/j.quaint.2017.06.059

Rios-Garaizar, J., 2012. Industria lítica y sociedad en la Transición del Paleolítico Medio al Superior en torno al Golfo de Bizkaia. PUbliCan - Ediciones de la Universidad de Cantabria, Santander.

Rios-Garaizar, J., 2017. A new chronological and technological synthesis for Late Middle Paleolithic of the Eastern Cantabrian Region. Quaternary International 433, 50–63. doi:10.1016/j.quaint.2016.02.020
