# Supplementary material for: A Middle Palaeolithic wooden digging stick from Aranbaltza III, Spain
Source: PLoS One. 2018 Mar 28;13(3):e0195044. doi: 10.1371/journal.pone.0195044 (PMC5874079; doi:10.1371/journal.pone.0195044)
Supplement: S3 File — (PDF) [file pone.0195044.s003.pdf]

S3. OSL dating

Sample preparation

In 2015, at the archaeological site of Aranbaltza OSL samples were collected by hammering PVC tubes (5x20 cm) into the freshly cleaned profiles. The material at the ends of each tube (~3 cm deep) was removed under dark room conditions to prevent light contamination during sampling. The material not exposed to light was wet-sieved, treated with concentrated HCl and H_2O_2 to remove carbonate and organic matter, respectively. Two steps of heavy liquid separation at 2.62 g·cm^{-3} and 2.72 g·cm^{-3} were carried out to remove the feldspar fraction and heavy minerals, respectively (Aitken, 1985; 1998). A magnetic separator was used to remove magnetic minerals from the samples following the protocol described in Porat, 2006.

The quartz-rich fraction was then etched for 40 minutes using 48% HF to remove any remaining feldspars and etch away the outer alpha irradiated layer. Following etching, the quartz fraction was treated with concentrated HCl for 60 minutes to remove any possible precipitated fluorides. After that, the sample was treated with a sodium pyrophosphate solution in an ultrasonic bath during 30 minutes, and washed several times to remove micas from the quartz-rich fraction.

For this study, a total of four luminescence samples were collected from the Aranbaltza III section. All of them yielded a good amount of quartz-rich fraction (more than 1 g). For single grain measurements, the quartz grains were mounted on aluminum discs with 100 holes with a 0.3 mm diameter.

The environmental dose rate determination was performed using a field gamma spectrometer (Canberra InSpector 1000) equipped with a NaI(Tl) probe, except for sample AZ15OSL-03 where the dose rate was obtained by high resolution gamma spectrometry using a High Purity Germanium Detector (HPGD). This sample was ground and packed into a gamma cup (Ø = 75 mm) and stored for four weeks to allow ^{222}Rn to build up equilibrium with ^{226}Rn before the measurements. The concentration values obtained were converted to dry beta and gamma dose rates using the conversion factors given in Guérin et al. (2011). Water contents for age calculation were between 24.1 % and 26.1 % in all of the samples. An internal quartz dose rate of 0.02 Gy·ka^{-1} (Vandenberghe et al., 2007) and a contribution from cosmic rays (Prescott and Hutton, 1994) were also incorporated into the total dose rate. The total environmental dose rates are listed in S3 Table 1.

Equipment and methods

Optical stimulation was carried out on a Risø TL/OSL Reader Model DA20 with a single grain attachment (green laser 532 nm; 10 mW). Luminescence was recorded using a photomultiplier tube (9235QB15), equipped with a 7mm Hoya-U340 filter. Samples were irradiated using a calibrated ^{90}Sr/^{90}Y source incorporated in the reader, with an effective dose rate of 0.11 ± 0.01 Gy·s^{-1}.

The natural OSL signals from the four samples listed in S3 Table 1 have been measured using the protocol given in S3 Table 2. In this work, we used the single aliquot regenerative (SAR) dose protocol adapted for single grain (Murray and Wintle, 2003; Wintle and Murray, 2006).

References

Aitken, M.J., 1985. Thermoluminescence Dating. Academic Press, London. 359 pp.

Aitken, M.J., 1998. An Introduction to optical dating: the dating of quaternary sediments by the use of photon-stimulated luminescence. Oxford University Press, Oxford. 267 pp.

Duller, G.A.T., 2003. Distinguishing quartz and feldspar in single grain luminescence measurements. Radiation Measurements 37, 161-165.

Guérin, G., Mercier, N., Adamiec, C., 2011. Dose-rate conversion factors: update. Ancient TL 29, 5-8.

Murray, A.S., Wintle, A.G., 2003. The single aliquot regenerative dose protocol: potential for improvements in reliability. Radiation Measurements 37, 377-381.

Porat, N., 2006. Use of magnetic separation for purifying quartz for luminescence dating. Ancient TL, Vol. 24 No. 2, 33-36.

Prescott, J.R., Hutton, J.T., 1994. Cosmic ray contributions to dose rates for luminescence and ESR dating: large depths and long-term time variations. Radiation Measurements 23, 497-500.

Vandenberghe, D., De Corte, F., Buylaert, J.-P, Kučera, J., Van den haute, P., 2008. On the internal radioactivity in quartz. Radiation Measurements 43, 771 – 775.

Wintle, A.G., Murray, A.S., 2006. A review of quartz optically stimulated luminescence characteristics and their relevance in single-aliquot regeneration dating protocols. Radiation Measurements 41, 369 – 391.

Table 1 Summary of the OSL samples from the Aranbaltza archaeological site used inthis study, listing the sample names, location within the stratigraphic unit, depth of the sample from the surface, assumed life-time average water content (10% error), total environmental dose rate, measured natural dose (D_e), number of accepted data in each sample, Mean Age of the different probability density areas (showed in S3 Figure 1), and Minimum Age Model and data overdispersion.

Sample Nº	Unit	Dosis (Gy·ka^{-1})	D_e (Gy)	Data (n)	Mean age (ka)	Minimum Age Model (ka)	Dispersion (%)
AZ15OLS-06	Unit 1	0.55±0.09	45.8±2.6	45	82.6±9.1	52.8±6.7	33.0±0.7
AZ15OSL-05	Unit 2	0.55±0.09	36.5±1.8	17	66.1±7.1	48.4±6.9	32.4±1.3
			63.5±10.3	4	115.0±21.6		
AZ15OSL-04	Unit 4	0.58±0.09	41.9±2.1	9	71.9±7.5	70.0±8.4	33.3±1.2
			80.1±4.6	15	137.5±14.8		
AZ15OSL-03	Unit 6	0.66±0.02	38.2±2.2	12	58.0±3.9	58.5±5.1	30.0±0.6
			75.0±3.0	33	113.8±6.0		

S3 Table 2. Single-aliquot regenerative-dose protocol (SAR) measurement conditions used in this study. A series of regenerative-dose (L_i) and test dose (T_i) OSL measurements are performed on each individual quartz grain to obtain a sensitivity-corrected dose–response curve on to which the sensitivity-corrected natural (L_n/T_n) signal is interpolated to obtain a D_e value. In order to check for contamination of the quartz OSL by feldspar grain or inclusions, a repeat dose point is added (step 1b), which includes an infrared (IR) bleach performed for 20 s at 50°C prior to the main L_i measurement, as described in Duller (2003). Step 1a is omitted when measuring a natural signal. Step 1b is added only when measuring the OSL IR depletion ratio (Duller, 2003).

Step	
**1a**	Give dose (L_d)
**1b**	IRSL (50ºC, 20 s)
**2**	Preheat (220ºC, 10 s)
**3**	Single Grain OSL (125ºC, 2 s) L_n or L_i
**4**	Test dose (10 Gy) T_d
**5**	Cutheat (200ºC, 10 s)
**6**	Single Grain OSL (125ºC, 2 s) T_n or T_i
**7**	TL bleaching (260ºC, 40 s)

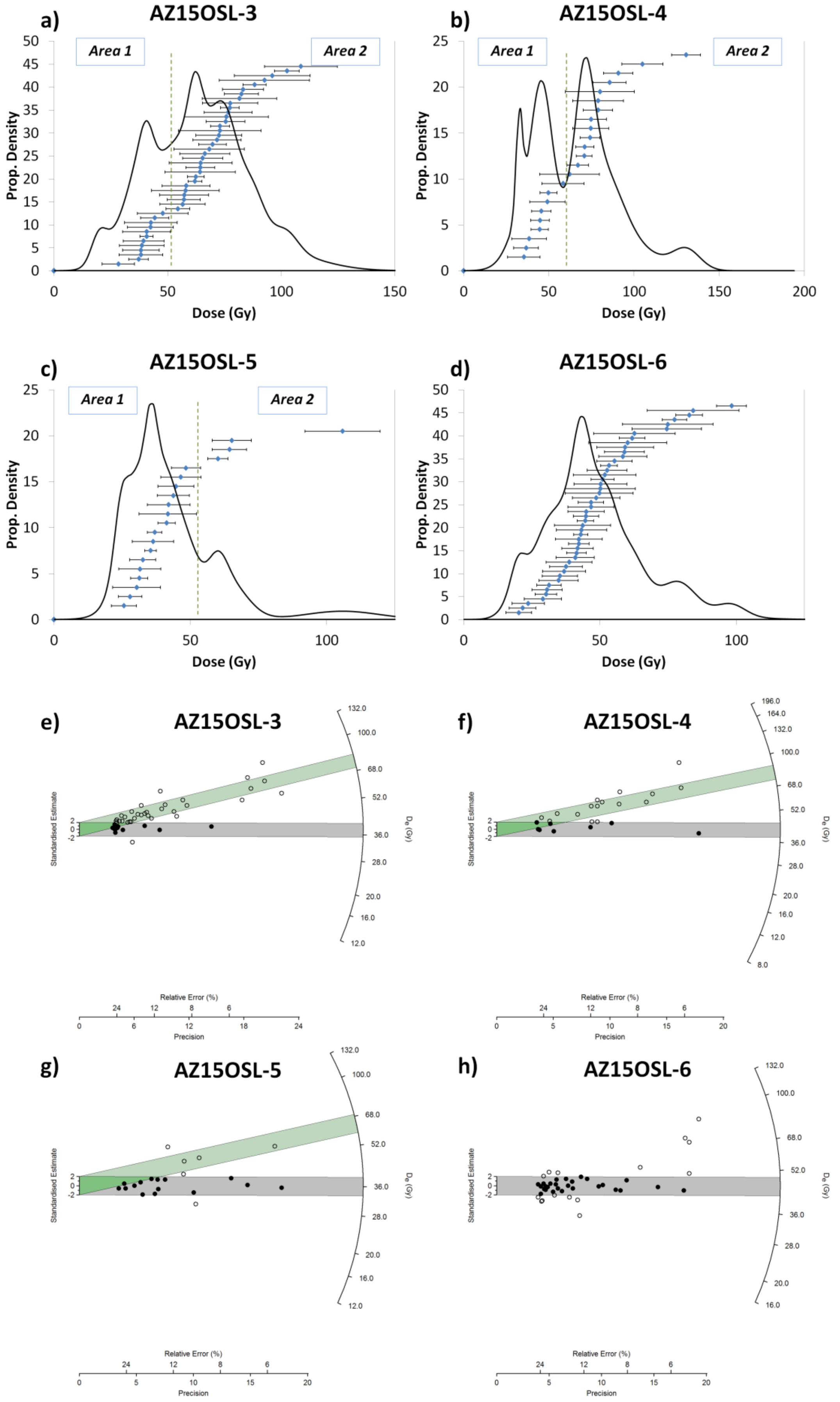

Figure 1. Probability density graphs of the different samples studied a) AZ15OSL-3, b) AZ15OSL-4, c) AZ15OSL-5 and d) AZ15OSL-6, and radial plots, e) AZ15OSL-3, f) AZ15OSL-4, g) AZ15OSL-5 and h) AZ15OSL-6. Dashed line

in a), b) and c) separate regions (Area 1 and Area 2) of different age characteristics. The grey and green shaded regions on the radial plots (e-g) are centered on the mean De values of Area 1 and Area 2 respectively.
