# Supplementary figures and images for: A Middle Palaeolithic wooden digging stick from Aranbaltza III, Spain
Source: PLoS One. 2018 Mar 28;13(3):e0195044. doi: 10.1371/journal.pone.0195044 (PMC5874079; doi:10.1371/journal.pone.0195044)

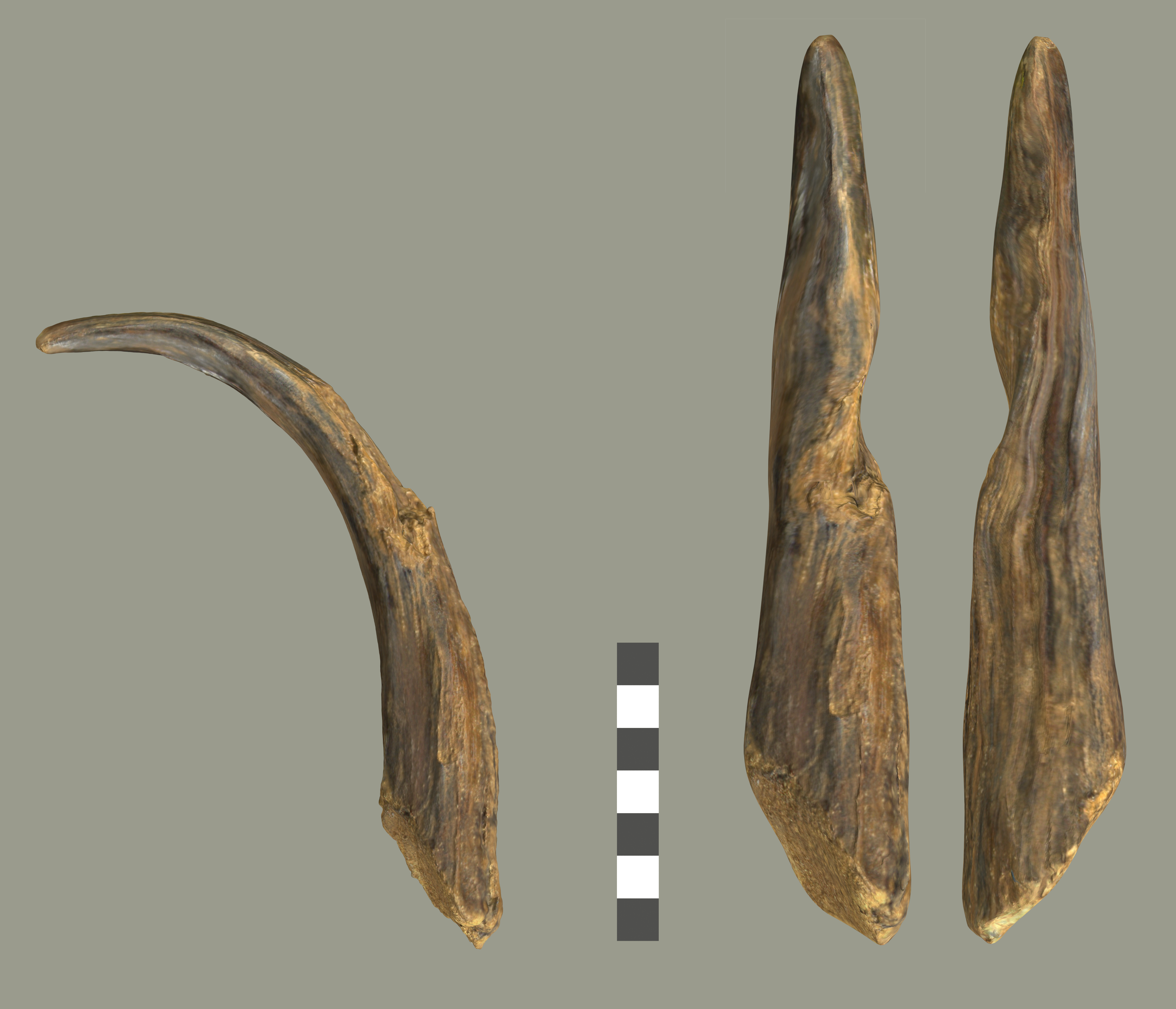

Supplement: S4 File — Virtual reconstruction of the pointed tool (right) obtained from 3D model of the piece at its current condition (left). (TIFF) [file pone.0195044.s004.tiff]

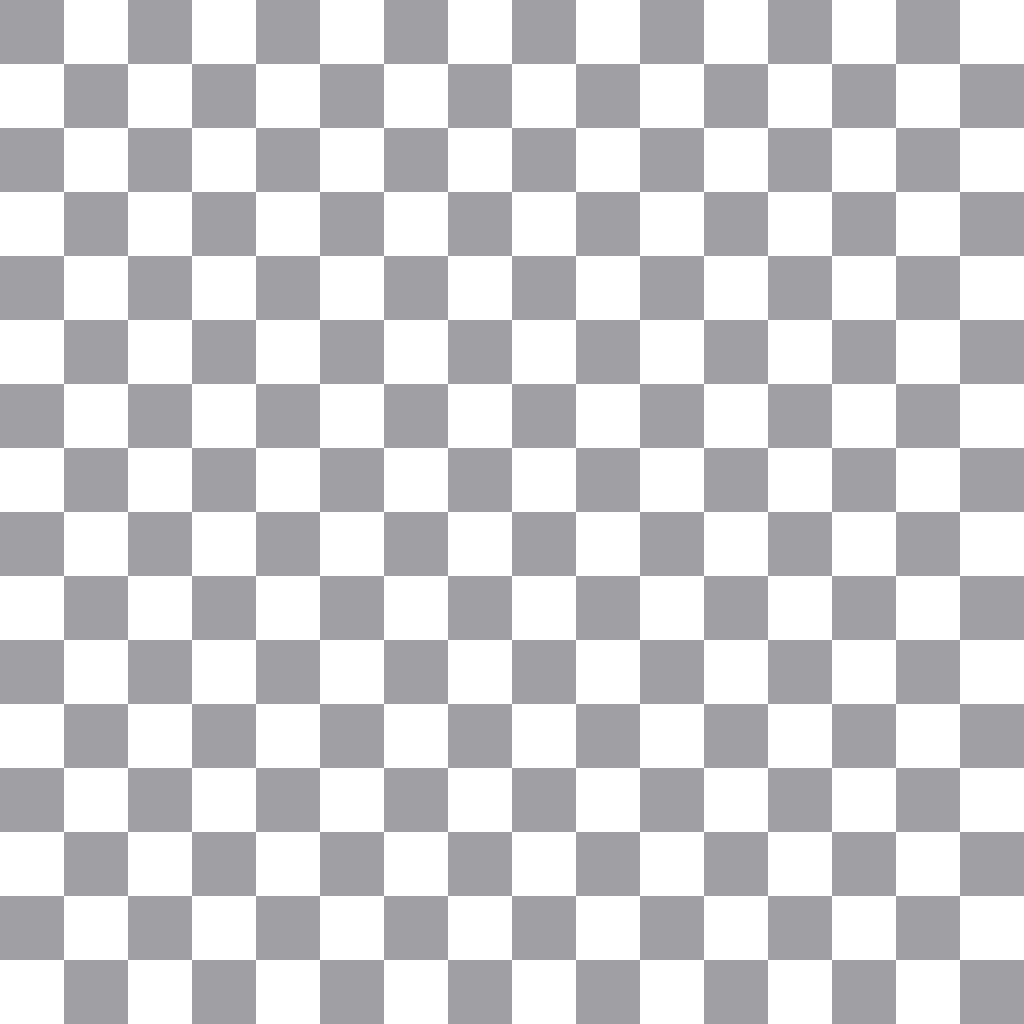

Supplement: S6 File — (ZIP) [file pone.0195044.s006.zip › 0_SFusion.png]
